# The Effects of Abortion Decision Rightness and Decision Type on Women’s Satisfaction and Mental Health

**DOI:** 10.7759/cureus.38882

**Published:** 2023-05-11

**Authors:** David C Reardon, Katherine A Rafferty, Tessa Longbons

**Affiliations:** 1 Research, Elliot Institute, St. Peters, USA; 2 Research, Charlotte Lozier Institute, Arlington, USA; 3 Psychology, Iowa State University, Ames, USA

**Keywords:** coerced abortion, unwanted abortion, abortion, mental health, reproductive rights, unsafe abortions, pregnancy loss, health policy, post-abortion adjustments, post-abortion mental health

## Abstract

Background

A case series report based on the Turnaway Study has previously concluded that 99% of women with a history of abortion will continue to affirm satisfaction with their decisions to abort. Those findings have been called into question due to a low participation rate (31%) and reliance on a single yes/no assessment of decision satisfaction.

Aim

To utilize more sensitive scales in assessing decision satisfaction and the associated mental health outcomes women attribute to their abortions.

Method

A retrospective survey was completed by 1,000 females, aged 41-45, living in the United States. The survey instrument included 11 visual analog scales for respondents to rate their personal preferences and outcomes they attributed to their abortion decisions. A categorical question allowed women to identify if their abortions were wanted and consistent with their own values and preferences, inconsistent with their values and preferences, unwanted, or coerced. Linear regression models were tested to identify which of three decision scales best predicted positive or negative emotions, effects on mental health, emotional attachment, personal preferences, moral conflict, and other factors relevant to an assessment of satisfaction with a decision to abort.

Results

Of 226 women reporting a history of abortion, 33% identified it as wanted, 43% as accepted but inconsistent with their values and preferences, and 24% as unwanted or coerced. Only wanted abortions were associated with positive emotions or mental health gains. All other groups attributed more negative emotions and mental health outcomes to their abortions. Sixty percent reported they would have preferred to give birth if they had received more support from others or had more financial security.

Conclusions

Perceived pressure to abort is strongly associated with women attributing more negative mental health outcomes to their abortions. The one-third of women for whom abortion is wanted and consistent with their values and preferences are most likely over-represented in studies initiated at abortion clinics. More research is needed to understand better the experience of the two-thirds of women for whom abortion is unwanted, coerced, or otherwise inconsistent with their own values and preferences.

## Introduction

A 2015 study undertaken by an abortion advocacy group, Advancing New Standards in Reproductive Health (ANSIRH), reported that 99% of women who had undergone abortion three years earlier answered yes to the question: “Given your situation, was the decision to have an abortion the right decision for you?” [1,2]. These findings were interpreted by ANSIRH as evidence of nearly universal “satisfaction with the abortion decision” and widely reported by mass media outlets as evidence that women seldom experience regrets or mental health issues following abortion [3]. But in a separate analysis of the same sample of women, ANSIRH elsewhere reported high levels of regret (41-66%), sadness (64-74%), guilt (53-63%) and anger (31-43%) [4]. This incongruency between high rates of negative feelings and the reported 99% “decision satisfaction,” as the findings were described by the authors, invited considerable criticism of both ANSIRH’s methodology and their sample’s representativeness [1,3,5,6]. Concerns over the accuracy and interpretation of these results were further heightened by ANSIRH’s refusal to share their research instruments for review or their data for reanalysis [5].

A chief methodological criticism was that ANSIRH’s binary yes/no question lacked a scale for identifying the degree of “decision satisfaction” [3]. In addition, the question preface (“Given your situation”) may have fixated responses on beliefs and feelings at the time of the abortion. Aside from the risk of inviting reaction formation, a response of “yes” to the ANSIRH question may have meant nothing more than an affirmation that respondents tried to make the “right decision” given their situation at that time. In such cases, it would not actually inform us if women believed their abortions improved their lives, much less if their experience was free of any regrets, guilt, nightmares, depression, suicidal thoughts, substance use, rapid repeat pregnancy, or any other negative effects which research has shown to be associated with abortion [5,7-10].

Another major criticism of ANSIRH’s decision rightness analyses relied on their use of a non-representative sample of women in their longitudinal case series branded as the Turnaway Study [3]. The invitations to participate were non-random. Moreover, only 31% of the women invited to participate in the ANSIRH survey completed at least one interview and half of that fraction dropped out prior to the last interview [3,11]. The poor participation rate is further highlighted in contrast to another ANSIRH study for which 72% of women seeking an abortion participated, though notably this latter study only requested women to complete a pre-abortion questionnaire; therefore, invitees did not face any anticipation of anxieties regarding an interview to discuss their post-abortion feelings [12]. It seems likely that the low 31% participation rate in ANSIRH’s decision rightness sample reflects a high degree of selection bias. This conclusion is consistent with the findings of studies that have found that women who anticipate the most negative reactions to their abortions are least likely to agree to participate in follow-up interviews when invited to do so at an abortion clinic [5,13,14]. Moreover, in a previous analysis of the present retrospective survey, we reported a 91% completion rate among women who had abortions after the topic of abortion was revealed [15]. This closely matches the 92% participation rate of a study regarding emotional adjustments following prophylactic mastectomies [16]. This suggests that retrospective studies initiated after an abortion has been completed, and not in association with the abortion clinic itself, may provoke less stress and therefore higher participation rates.

While ANSIRH’s effort to invite women to offer a post hoc evaluation of the “rightness” of their abortion decision is not without merit, answers to this question should have been evaluated in the context of other measures of benefits or harm women attribute to their abortion experience. This is important because many studies have revealed that negative and positive reactions frequently co-exist [5]. While that fact was recognized in ANSIRH’s own analyses, they concluded that decision rightness and emotional adjustment are not significantly correlated, writing “Believing abortion was the wrong decision and experiencing negative emotions are distinct…,”, a conclusion that is at odds with our own research and the self-reports of women [1,5].

In addition, ANSIRH’s researchers and other proponents of unrestricted abortion generally analyze and interpret their findings from the perspective that women only seek abortion for “unwanted pregnancies” despite consistent evidence that a substantial percentage of women are aborting pregnancies that were planned or welcomed, often due to pressure to abort from others or circumstances [5,15,17-19]. For example, analyses of the National Longitudinal Survey of Adolescent to Adult Health revealed that approximately 20% of women admitting a history of abortion reported that one or more of their aborted pregnancies had been wanted [20]. In addition, the same study found that abortion of wanted pregnancies was significantly associated with higher rates of subsequent psychological disorders. Those findings are consistent with the American Psychological Association’s 2008 task force report which found that negative reactions to abortion were more common for women “terminating a pregnancy that is wanted or meaningful” or when there is “perceived pressure from others” [5,21].

In light of the above issues, the goal of the present study is to improve on the assessment and understanding of decision rightness, decision types, and decision satisfaction utilizing more nuanced scales and a more random and representative sample of women than was utilized in ANSIRH’s Turnaway Study. An additional goal is to understand how assessments of decision rightness correlate to other measures applicable to assessing decision satisfaction and the mental health adjustments associated with abortion. Regarding these other measures, we hypothesized that differences in the abortion decision scale and a related decision type scale would be strongly correlated with the degree of self-reported moral and/or maternal conflicts, positive and/or negative emotional reactions, and the direction of mental health effects that women self-attribute to their abortions.

## Materials and methods

Experts in abortion and mental health research were consulted in preparing a questionnaire for our Unwanted Abortion Studies, a series of investigations into the prevalence and effects of abortions that conflict with women’s own maternal preferences and moral beliefs. Employing the survey panel services of Cint.com, we collected 1,000 completed surveys from females who are residents of the US and 41 to 45 years of age, both inclusive. Cint panelists are persons who voluntarily complete surveys using their own electronic devices in exchange for small rewards with a value under $3. The Cint survey panels include over 28 million US residents. A narrow age range was chosen to eliminate the confounding effects of age while capturing the experience of women who have completed the majority of their reproductive lives. Additional details about the sample were previously published in an analysis of pressures to choose abortion [15]. Notably, in that previous study, we found that the demographic characteristics of the subgroup of women who reported abortions may somewhat underrepresent women who are less educated, less affluent, and Black, compared to the distribution rates reported elsewhere for women in these subgroups [15,22,23].

The questionnaire included 11 visual analog scales shown in Table 1. For each scale respondents were shown a horizontal line with a slider they moved to show the range of their agreement or disagreement relative to the two labels at either end. Responses were electronically coded from zero to 100, resulting in a scale range of 101 points. Among these items, ANSIRH’s central research question was reframed as the statement, “Given my situation, the decision to have an abortion was the right decision for me.” This allowed respondents to provide a range of agreement from “Not at all” to “Very much so,” rather than simply yes or no.

**Table 1:** Survey Scales, abbreviations and range labels (0 to 100)

Abbreviation	Complete statement or question	Scale of Agreement
RightDecision	Given my situation, the decision to have an abortion was the right decision for me.	Not at all true | Very true
PersonalPref	Excluding the pressures I faced to have an abortion, in terms of satisfying my own personal preferences the abortion was . . .	Very unwanted | Very wanted
MoreSupport	If I had received more support from others, I would have continued the pregnancy.	Not at all true | Very true
MoreFinSecurity	If I had more financial security, I would have continued the pregnancy.	Not at all true | Very true
MoralConflict	The idea of abortion conflicted with my maternal desires.	Not at all | Very much so
MaternalConflict	The idea of abortion conflicted with my moral beliefs.	Not at all | Very much so
EmotionalAttachment	My emotional attachment to the pregnancy was...	None at all | Very high
HumanLife	I perceive the pregnancy as being . . .	A clump of cells | A human life
PositiveEmotions	My positive emotions regarding the abortion are . . .	None at all | Very high
NegativeEmotions	My negative emotions regarding the abortion are . . .	None at all | Very high
BetterMentalHlth	Abortion made my mental health . . .	Very much worse | Very much better

An additional categorical question was asked: “Which best describes your abortion decision?” Respondents were presented with four possible answers: “Wanted and consistent with my values and preferences,” (Wanted), “Accepted but inconsistent with my values or preferences” (Inconsistent), “Unwanted and contrary to my values and preferences” (Unwanted) or “Coerced and contrary to my values and preferences” (Coerced). For parametric analyses, these categorical responses were recoded from 1 through 4 from Wanted, Inconsistent, Unwanted, and Coerced, respectively.

Three additional variables were calculated for this analysis. The first was an assessment of the more dominant trend in their emotional response to their abortions (NetEmotions), calculated by subtracting the score for NegativeEmotions from PositiveEmotions, yielding a possible range from -100 to +100. BetterMentalHlth was recoded using the formula 2*(BetterMentalHlth-50), yielding a range from -100 to +100, and assigned to a variable for mental health effects (MHeffects) with the sign and value representing both the direction (negative or positive) and degree of the effect women attributed to their abortions. Third, we recoded RightDecision scores below and above 50 to RightD2 as a zero or one, respectively, in order to approximate the equivalent of a no or yes answer to ANSIRH’s original question.

Finally, three univariate linear regression models, separately utilizing RightDecision, RightD2, and DecisionType as independent variables, were run for each of the dependent variables and were tested for best fit using Akaike information criterion (AIC).

The authors assert that all procedures contributing to this work comply with the ethical standards of the relevant national and institutional committees on human experimentation and with the Helsinki Declaration of 1975, as revised in 2008. All procedures involving human subjects/patients were approved by Sterling Institutional Review Board issued (ID:10225). Consent for survey participation, without prior notice of the topic, was digitally obtained from all respondents by Cint.com. No information was collected that would allow the authors to identify individual participants. Analyses were conducted using RStudio (Build 576; Posit, Boston, MA).

## Results

To obtain a total of 1,000 completed surveys, a total of 1,161 persons identified by Cint to be females in our age range responded to a survey invitation that did not reveal the topic. The first two pages contained only demographic questions which were used to disqualify 122 respondents based on their self-reported age or gender. Of the remaining 1,039 respondents, 248 (23.7%) reported a history of abortion, which closely matches the Guttmacher Institute’s estimate that by the age of 45, 23.7% of American women will experience an induced abortion [22]. Of the 248 reporting a history of abortion, 226 (91%) completed the survey. Only the latter were included in the analyses.

Regarding DecisionType, 33% described their abortions as Wanted, 43% as Inconsistent, 14% as Unwanted and 10% as Coerced. In addition, 54% answered mostly affirmative (≥50) to the statement that they would have continued their pregnancy if they had more financial security, 42% would have given birth if they had more support from others, and 60% reported they would have preferred to give birth if they had received either more emotional support or had more financial security.

General descriptive statistics for scales, including the mean (M), standard deviation (SD), quartiles and the minimum and maximum responses are shown in Table 2. This table reveals that even while the mean of the RightDecision scale (75.55) was well above the centerpoint (50) the mean of all the other variables were either near the center or were negative.

**Table 2:** Descriptive statistics of variables Descriptive statistics of variables, including mean (M), standard deviation (SD), and quartiles

Label	M	SD	min	25%	median	75%	max
RightDecision	75.55	27.79	0	59	84	100	100
PersonalPref	54.46	31.97	0	33	52	80	100
MoreSupport	41.30	35.69	0	3	37	74	100
MoreFinSecurity	48.52	37.45	0	4	54	83	100
MoralConflict	49.11	34.79	0	19	51	76	100
MaternalConflict	46.32	35.08	0	9	50	76	100
EmotionalAttachment	48.81	31.63	0	21	49	76	100
HumanLife	52.55	34.55	0	20	51	83	100
PositiveEmotions	50.37	30.54	0	29	49	73	100
NegativeEmotions	50.65	32.97	0	23	51	78	100
NetEmotions	-0.28	55.65	-100	-39	0	36	100
BetterMentalHlth	49.03	23.37	0	35	50	61	100
MHeffect	-1.94	46.74	-100	-30	0	22	100

**Figure 1:**
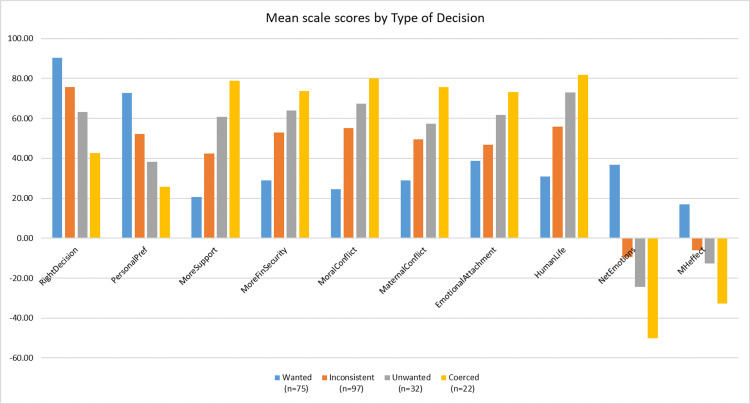
Mean scale scores disaggregated by DecisionType The image was created by the authors using Microsoft Excel.

Figure 1 shows the mean score for each scale segregated by the self-identified decision type groups: Wanted, Inconsistent, Unwanted, Coerced. In each case, the results revealed a consistent trend. Women whose abortions were wanted and consistent with their values and preferences reported the highest average score for RightDecision, PersonalPref, NetEmotions, and MHeffect. The three other groups were all more likely to attribute an overall negative effect on their mental health to their abortions, more negative than positive feelings, more moral and maternal conflicts over their abortion decision, less confidence in the rightness of their decision, less satisfaction with their decision as aligning with their own personal preferences, and were more likely to report that they would have given birth if they had received more support from others and/or had more financial security.

Table 3 shows the exact values for each data point shown in Figure 1, plus the additional variable, RightD2 which approximates the distribution of “yes” or “no” to the ANSIRH decision rightness question. RightD2 indicates that 94.7%, 89.7%, 62.5%, and 40.9% for the Wanted, Inconsistent, Unwanted, and Coerced groups, respectively, would most have answered “yes” if asked ANSIRH’s form of the question.

**Table 3:** Mean scale scores by DecisionType groups

	Wanted (n=75)	Inconsistent (n=97)	Unwanted (n=32)	Coerced (n=22)	Total (n=226)
RightD2	0.9466	0.8969	0.6250	0.4090	0.8274
RightDecision	90.25	75.70	63.22	42.68	75.55
PersonalPref	72.72	52.23	38.16	25.82	54.46
MoreSupport	20.57	42.35	60.88	78.82	41.30
MoreFinSecurity	28.88	52.91	64.00	73.64	48.52
MoralConflict	24.63	55.03	67.28	80.00	49.11
MaternalConflict	28.88	49.52	57.28	75.73	46.32
EmotionalAttachment	38.64	46.88	61.72	73.23	48.81
HumanLife	30.93	55.90	72.91	81.91	52.55
NetEmotions	36.69	-9.60	-24.38	-50.23	-0.28
MHeffect	16.91	-5.98	-12.63	-32.82	-1.94

Table 4 shows the correlation coefficients for every combination of the variables and reveals that all of these variables were significantly correlated to each other with p<.01 for all cases. The strongest correlation (.70) was between MaternalConflict and MoralConflict. There was also a strong correlation (.68) between MoreFinSecurity and MoreSupport, which suggests that in many cases the lack of support from others was linked to a perception that the other persons argued for the abortion due to financial considerations. The next strongest correlation (.65) was between EmotionalAttachment to the unborn child and MaternalConflict, which was also mirrored in a high correlation (.57) between the perception that the pregnancy involved a HumanLife and EmotionalAttachment.

**Table 4:** Correlation matrix of all variables with confidence intervals Values in square brackets indicate the 95% confidence interval for each correlation. The confidence interval is a plausible range of population correlations that could have caused the sample correlation. * indicates p < .05; ** indicates p < .01

Variable	1	2	3	4	5	6	7	8	9	10
1. TypeDecision										
2. RightDecision	-.51**									
	[-.60, -.40]									
3. PersonalPref	-.47**	.44**								
	[-.57, -.36]	[.32, .54]								
4. MoreSupport	.51**	-.47**	-.36**							
	[.41, .60]	[-.57, -.36]	[-.47, -.24]							
5. MoreFinSecurity	.39**	-.34**	-.30**	.68**						
	[.28, .50]	[-.45, -.22]	[-.41, -.17]	[.60, .74]						
6. MoralConflict	.52**	-.39**	-.30**	.58**	.45**					
	[.42, .61]	[-.49, -.27]	[-.42, -.18]	[.49, .66]	[.34, .55]					
7. MaternalConflict	.40**	-.36**	-.39**	.54**	.44**	.70**				
	[.29, .51]	[-.47, -.25]	[-.50, -.28]	[.44, .63]	[.33, .54]	[.62, .76]				
8. EmotionalAttachment	.34**	-.39**	-.40**	.49**	.35**	.50**	.65**			
	[.22, .45]	[-.50, -.28]	[-.50, -.28]	[.39, .58]	[.23, .46]	[.39, .59]	[.57, .72]			
9. HumanLife	.49**	-.39**	-.41**	.55**	.37**	.54**	.52**	.57**		
	[.39, .59]	[-.49, -.27]	[-.51, -.29]	[.45, .64]	[.25, .48]	[.44, .63]	[.42, .61]	[.48, .65]		
10. NetEmotions	-.49**	.50**	.53**	-.48**	-.55**	-.57**	-.54**	-.42**	-.47**	
	[-.59, -.39]	[.40, .59]	[.43, .62]	[-.58, -.37]	[-.63, -.45]	[-.65, -.48]	[-.62, -.44]	[-.52, -.31]	[-.57, -.36]	
11. MHeffect	-.32**	.43**	.40**	-.27**	-.28**	-.30**	-.33**	-.36**	-.38**	.59**
	[-.43, -.20]	[.32, .53]	[.28, .50]	[-.38, -.14]	[-.40, -.16]	[-.41, -.17]	[-.44, -.20]	[-.46, -.24]	[-.49, -.27]	[.50, .67]

For each outcome variable, three univariate linear regression models were constructed using DecisionType, RightDecision, and RightD2 as separate independent variables. AIC model selection was then used to identify which independent variable was the best-fit model for each outcome variable. RightD2, emulating ANSIRH’s binary variable, had the worst fit for every model tested. RightDecision had the best fit for three outcome variables: EmotionalAttachment, NetEmotions, and MHeffect. DecisionType was the best fit for all other outcome variables.

## Discussion

Our findings revealed that only one in three women described their abortions as both wanted and consistent with their own values and preferences. Two-thirds experienced their abortion decision as a violation of their own values and preferences, with 24% describing their abortions as unwanted or coerced. A majority of women who had abortions (60%) reported they would have carried to term if they had received more support from others and/or had more financial security. Both factors indicate that abortion is a marginal, or even unwanted, choice for most women. These findings are consistent with the results of other investigations reporting high rates of perceived pressure to abort and ambivalence regarding abortion decisions [24-28].

Overall, only women who describe their abortion choice as wanted and consistent with their own values and preferences attributed any mental health benefits or a net gain in positive emotions to their abortions. All other groups attributed more negative emotions and a decline in mental health to their abortions. For these other groups, more social support, both from individuals and society, especially in terms of financial assistance, might empower those women who are at greatest risk of unwanted abortions to make choices more in line with their own personal values and preferences.

ANSIRH’s studies predicted that 99% of women with a history of abortion would affirm that, given their individual situations, abortion was the right choice [1]. In our sample, however, when the RightDecision scale was converted to a binary RightD2 (simulating ANSIRH’s binary yes or no decision assessment) only 82.7% mostly agreed with the statement that abortion was the right decision.

Greater insight is obtained, however, when RightDecision is segregated by our DecisionType variable. That segregation reveals that the Wanted group, for whom the abortion choice was consistent with their own values and preferences, was most similar to ANSIRH’s sample, with 94.7% agreeing (RightDecision≥50) that their decision was the right decision.

The observed disparity between ANSIRH’s sample and our own are most likely due to ANSIRH’s methodology. Previous studies have shown that women who anticipate negative feelings about their abortions are least likely to accept requests at abortion clinics for follow-up interviews [5,13,29]. This results in self-censure, with the women who are most prone to negative outcomes declining to participate. ANSIRH’s selection bias was further exacerbated by a non-random invitation process, which included total exclusion of women seeking abortions due to suspected fetal anomaly, a subgroup known to be at higher risk of more negative reactions [2,5]. Even with the incentive of a $50 gift card for each interview, only 31% of the women invited to participate in ANSIRH’s post-abortion survey completed at least one interview.

By comparison, our retrospective survey through Cint.com panels had a 91% completion rate with a cost of only $3 per completed interview [15]. Notably, in a pre-abortion survey conducted by ANSIRH, 70% of women asked to participate completed the in-clinic survey [12]. This is over double the participation rate of their post-abortion survey, the Turnaway Study. This higher participation rate was most likely possible because abortion patients were not asked to participate in a post-abortion study, which many likely perceived as a more stressful experience. This difference suggests that abortion clinic-initiated studies might obtain more representative samples of patients when post-abortion interviews are not required. It is likely that retrospective studies that are not connected with the abortion provider, such as ours, are associated with less stress and avoidance behaviors, especially for women who are being anonymously queried many years after their abortion experiences.

In short, our findings suggest that clinic-initiated surveys are likely to oversample women for whom the abortion decision is wanted and consistent with their own values and preferences and are likely to underrepresent, or even miss altogether, women for whom the abortion is unwanted or coerced, since the latter may be least likely to agree to follow-up interviews. Notably, the 31% participation rate in ANSIRH's Turnaway Study closely parallels the 33% of women in our sample who described their abortions as wanted and consistent with their values and preferences. In addition, our findings contradict ANSIRH’s hypothesis that decision satisfaction and emotional responses are not linked [1].

Another key finding of our study is that ANSIRH’s binary “decision rightness” question is clearly not representative of decision satisfaction. The majority of women in our sample who reported agreement (≥50) with the statement “Given my situation, the decision to have an abortion was the right decision for me,” elsewhere indicated a preference for having given birth rather than having an abortion. This is especially clear in the responses related to DecisionType, MoreSupport, and MoreFinSecurity. At least in part, the predicate phrase, “given my situation” in the ANSIRH question may have led many women to interpret the statement as equivalent to “I made the best decision I could at that time.” An affirmation of having made the best decision available to oneself does not imply, much less promise, satisfaction with that decision. In addition, even the phrase “right decision” invites ambiguity, both for respondents and the interpreters of these results. Was the decision “right” because it was the preferred choice, their most beneficial choice, the only available or even allowed choice (in cases of coercion and abuse), the right moral choice, a civil right, or merely “right” because the question triggers a reaction formation response leaning toward an affirmation of a past choice that cannot be changed? Future research should investigate each of these options, all of which reveal important nuances in women’s abortion choices and their retrospective evaluation of those choices.

In general, our findings reveal that DecisionType provides a better metric for gauging issues related to satisfaction or dissatisfaction with an abortion decision than RightD2, which was most similar to ANSIRH’s dichotomous measure. But our RightDecision scale provided a better linear regression fit than DecisionType for the variables EmotionalAttachment, NetEmotions, and MHeffect. This may be true because the 101-point RightDecision scale allowed for more sensitivity than our four categories for DecisionType. The latter might be improved by implementation on an analog sliding scale. Further study is necessary to determine if any single question regarding the abortion decision can provide the best model fit for predicting the relative benefits and risks that specific women are most likely to experience, given their own unique situations. Enough is already known to inform pre-abortion screening and counseling services in order to better counsel women who are at greatest risk of unwanted and unsafe abortions [30], but a greater focus on these issues is warranted both in research and clinical settings.

One strength of our study is that the total percentage of respondents reporting a history of abortion closely matches the expected rate for this age group [22]. In addition, compared to ANSIRH’s 31% completion rate of their first interview, our 91% completion rate for women reporting a history of abortion was very high. However, that 9% drop-out rate was still four times higher than that that of women without a history of abortion, suggesting that self-censure is likely to continue to bias results toward underreporting of negative effects even in prospective studies many years after exposure to an abortion [15]. Another limitation of our study is that Black women, low-income women, and lower educated women (groups who are likely at greater risk of feeling pressured to have an unwanted abortion) are also somewhat unrepresented when our sample is compared to the abortion rates of these groups reported elsewhere [15]. This factor, too, suggests that our results may underestimate both the true rate of unwanted and coerced abortions and their associated negative outcomes. Therefore, any projection of the rates of negative reactions and unwanted abortions on the national population are more likely to be underestimates than overestimates. In spite of these limitations, however, the correlations between the type of abortion decision and negative effects are likely to be accurate.

Another limitation is that our data is both retrospective and limited to one point in time. Various perceptions may change, or conversely, harden over time. For example, just as victims of sexual abuse may only later recognize how they had been manipulated and abused, it is possible that some portion of the women in our sample who report that they were coerced into their abortions may have perceived their choice as freely made at that time. Similarly, there is conflicting evidence regarding the course of negative emotions over time. One case-series study based on patients recruited at three abortion clinics reported a trend towards increased negative emotions over two years [31], while ANSIRH’s case series of similarly recruited patients reported a trend toward declining negative emotions [1]. But efforts to identify the differences in these finding have been blocked by both sets of authors through their refusal to provide any further details or findings beyond what they have chosen to publish or to share their data for reanalysis [5].

However, even if the trend in negative emotions could be reliably measured over the first one to five years after an abortion, case reports and other retrospective surveys have revealed that many women successfully repress negative emotions for many years, even decades [32-34]. For example, one survey of women who sought post-abortion counseling revealed that 63% reported a period of time (averaging over five years) during which they successfully denied or repressed negative feelings and doubts about their abortions [33]. Notably, for many, the successful repression of negative thoughts is often broken by some specific triggering event such as the death of a loved one, a miscarriage, or the birth of a later child [32-34]. This underscores the difficulty in attempts to measure the frequency of negative reactions facing every study design. Some women experience the bulk of their negative reactions immediately, while many (perhaps most), begin to experience negative reactions years or even decades later. Moreover, it is clear that many women who do experience negative outcomes that they attribute to their abortions often receive counseling, medication, or natural healing over time [5]. Any of these mitigating factors would dramatically reduce the degree of negative emotions that would be reported in survey responses at any specific time. This point is especially important in regard to interpreting the results of studies that employ standardized scales. For example, the ANSIRH studies employed the Brief Symptom Inventory, which asks respondents to indicate the degree, if any, of symptoms of depression or anxiety that they experienced in the seven days prior to their interview [35]. But clearly, the rate of women reporting abortion associated depression in the last seven days prior to an interview will always be far lower than the rate reported by women who were asked if they had ever experienced depression, which they attributed to their abortions. In short, while the retrospective nature of our study design introduces important limitations on the interpretation of our results, it also introduces the advantage of allowing the participants to report on their emotional and mental health experiences overall rather than just in the last seven days.

Still, we recommend that future studies should include both long-term self-assessments of symptoms women attribute to their abortion experiences alongside standardized mental health scales. The latter were not employed for this study in order to simplify the survey, reduce its length, and to reduce obstacles in the way of completing the survey. Also, while the present study was focused on how the decision rightness scales and decision type variable correlate to decision satisfaction and well-being, additional research must be done to understand better how a variety of these factors, such as moral conflict and lack of sufficient financial resources, impact mental health and decision satisfaction. Similarly, previous research has indicated that socially-based and internally-based conflicts may provide separate paths to negative emotions following an abortion [36]. The survey tools used in the present investigation may be successfully deployed to deepen our understanding of those differences.

Ideally, more prospective longitudinal studies should be undertaken which include data on prior mental health, pregnancy intention, and other confounding factors years prior to the participants’ first pregnancies. Unfortunately, while a few high quality prospective studies have been done, the underlying data gathered for these studies was general in nature: The questionnaires were not designed to focus on research questions specific to the abortion experience [8,20,37]. Therefore, we recommend that new and existing national prospective survey designs should include input from experts on both sides of the abortion and mental health controversy to ensure better that the most useful questions are included. Ideally, the full range of interactions between reproductive health experiences including abortion, natural losses, infertility, postpartum adjustments, newborn disabilities, and other interactions between these reproductive experiences, mental health, and socioeconomic well-being would be addressed in a dedicated longitudinal study, like that which was recommended by Surgeon General C. Everett Koop fully 34 years ago [33]. Better research tools will lead to greater clarity about the post-abortion experience and the needs of women exposed to unwanted abortions.

## Conclusions

ANSIRH’s dichotomous, yes-or-no assessment of decision rightness was too blunt of an instrument to properly assess women’s satisfaction with their abortions. Both our 101-point scale for rating decision rightness and our categorical scale for identifying the type of decision (Wanted, Inconsistent, Unwanted, or Coerced) provided strong correlations to measures related to women’s satisfaction with their abortion experiences. In addition, our findings suggest that ANSIRH’s non-random sampling method, further compromised by a 69% refusal to participate rate, most likely lacks sufficient representation of the majority of women for whom the abortion choice is inconsistent with or violates their own values and preferences.

Our findings indicate, as a conservative estimate, that two-thirds of women experienced their abortions as a violation of their own values and preferences. A majority of women who had abortions (60%) reported they would have carried to term if they had received more support from others or had felt more financial security, and one-fourth described their abortions as either unwanted or coerced. On average, only women who described their abortions as wanted and consistent with their values and preferences (33%) attributed any benefits to their abortions. All other groups were more likely to attribute an increase in negative emotions and a decline in mental health to their abortions, report more stress when questioned about their abortion experiences, and appear less likely to participate in surveys initiated at abortion clinics as compared to women for whom the abortion is wanted and consistent with their values and preferences.

More research is needed to investigate the factors involved in abortion decisions and how these interact with both positive and negative outcomes. The finding that our simple four-point categorical scale for distinguishing between abortions that are freely wanted, accepted, unwanted, or coerced is strongly correlated with more positive or negative outcomes should be of special interest to mental health professionals and could be used as a starting point when called upon to advise pregnant patients on their abortion decisions. This scale could also be used as a guide to identifying issues that may need to be discussed when treating patients who are experiencing grief, guilt or other issues they attribute to their abortions.

## Appendices

**Data availability statement:** The data that support the findings of this study are available from the Mendeley depository at http://dx.doi.org/10.17632/5hgj345svc.1 but are embargoed until October 1, 2023 in order to provide the authors with additional time to complete and publish additional analyses.
